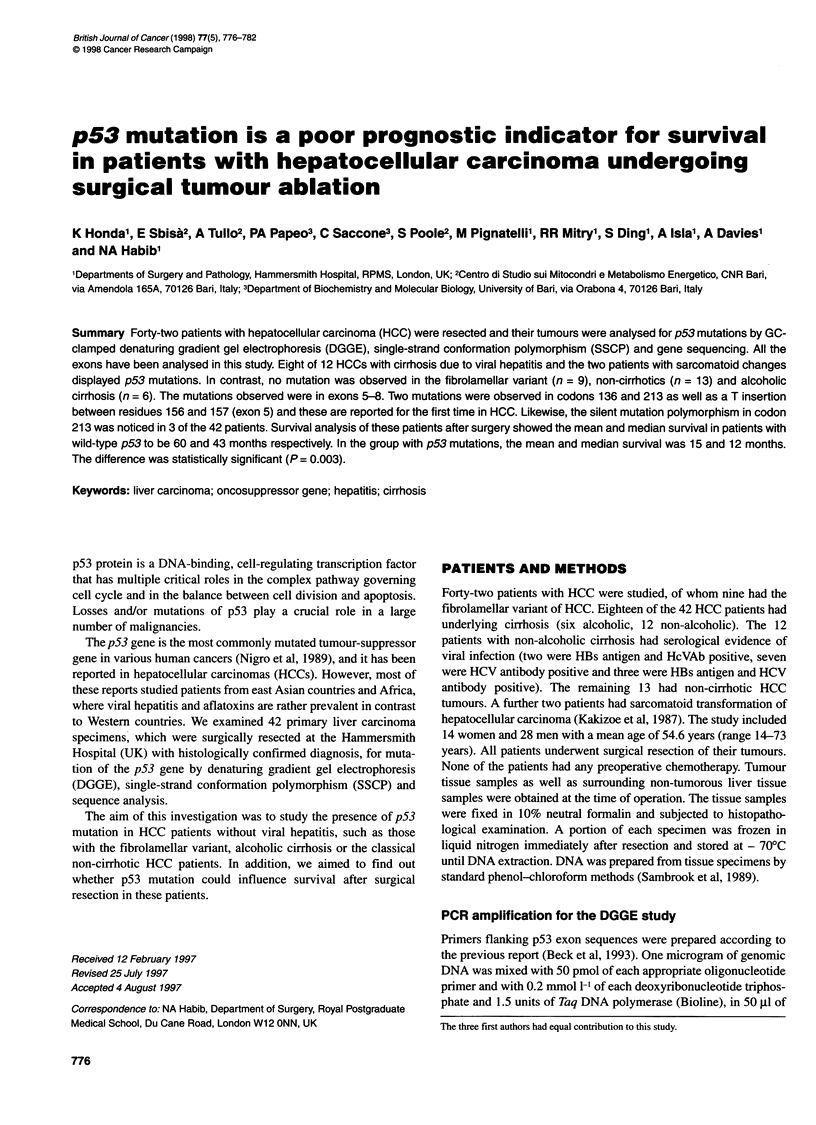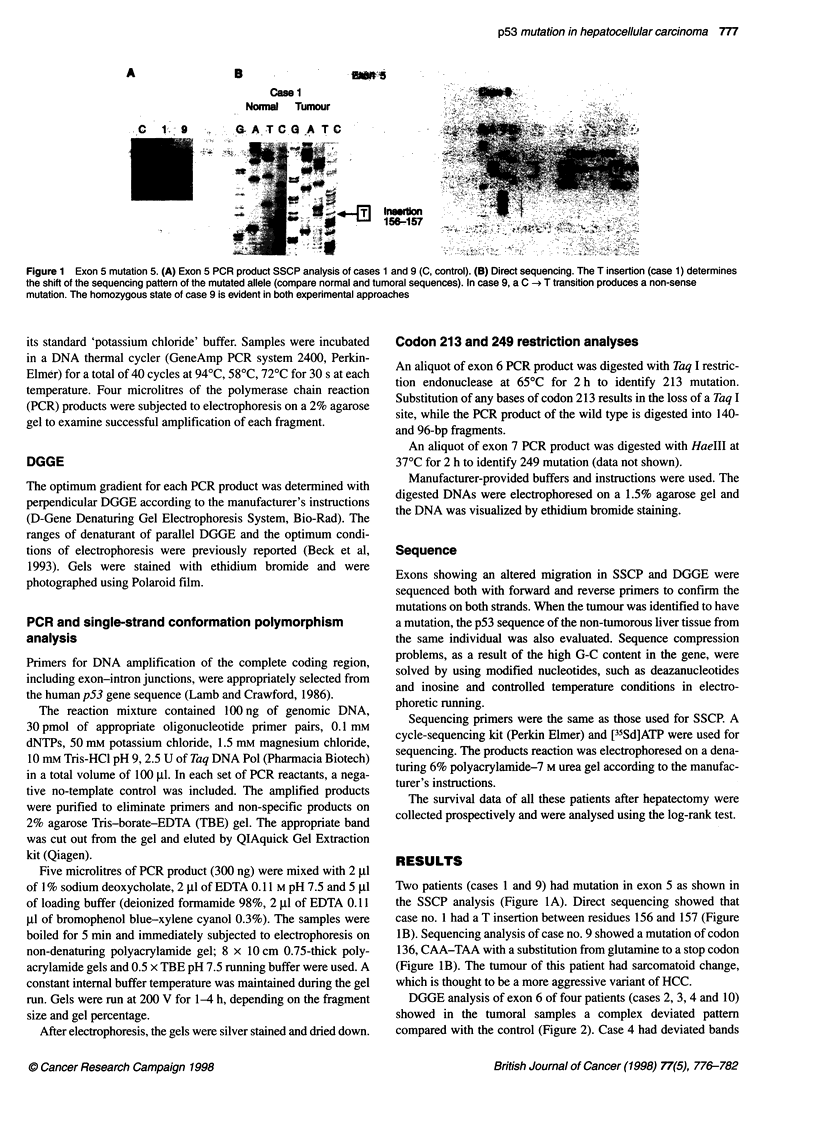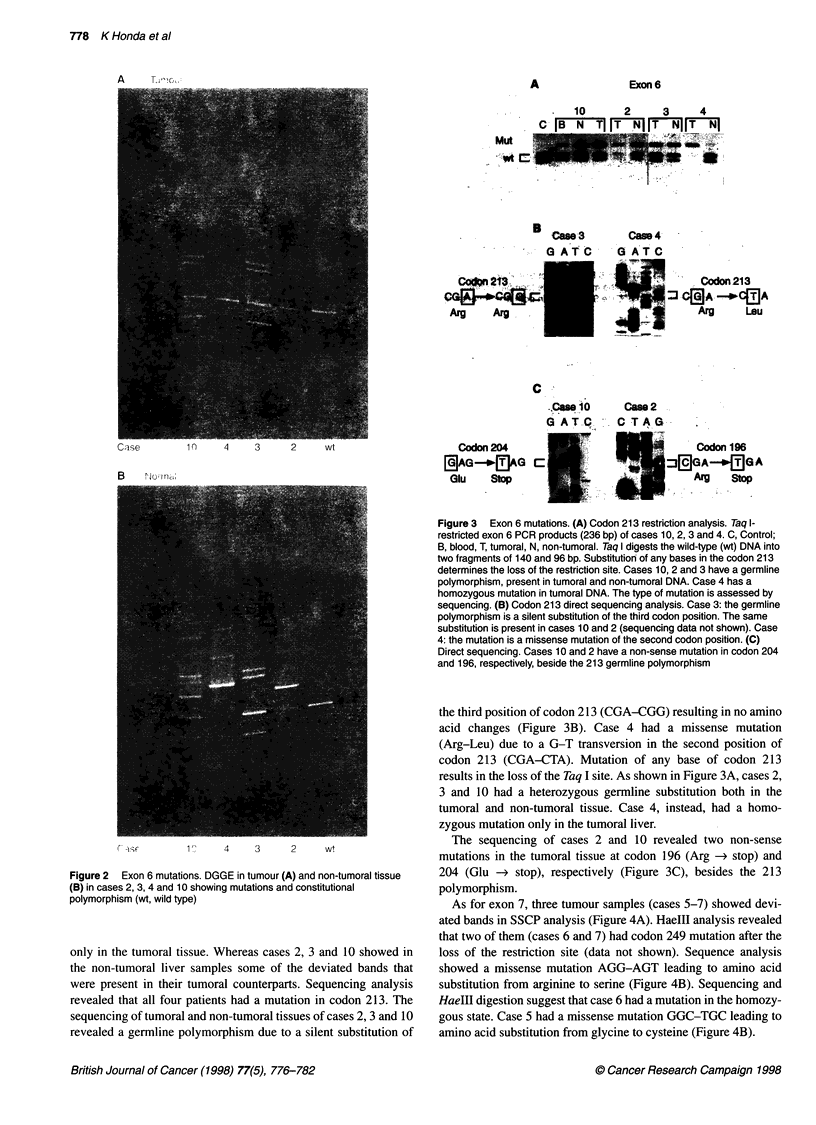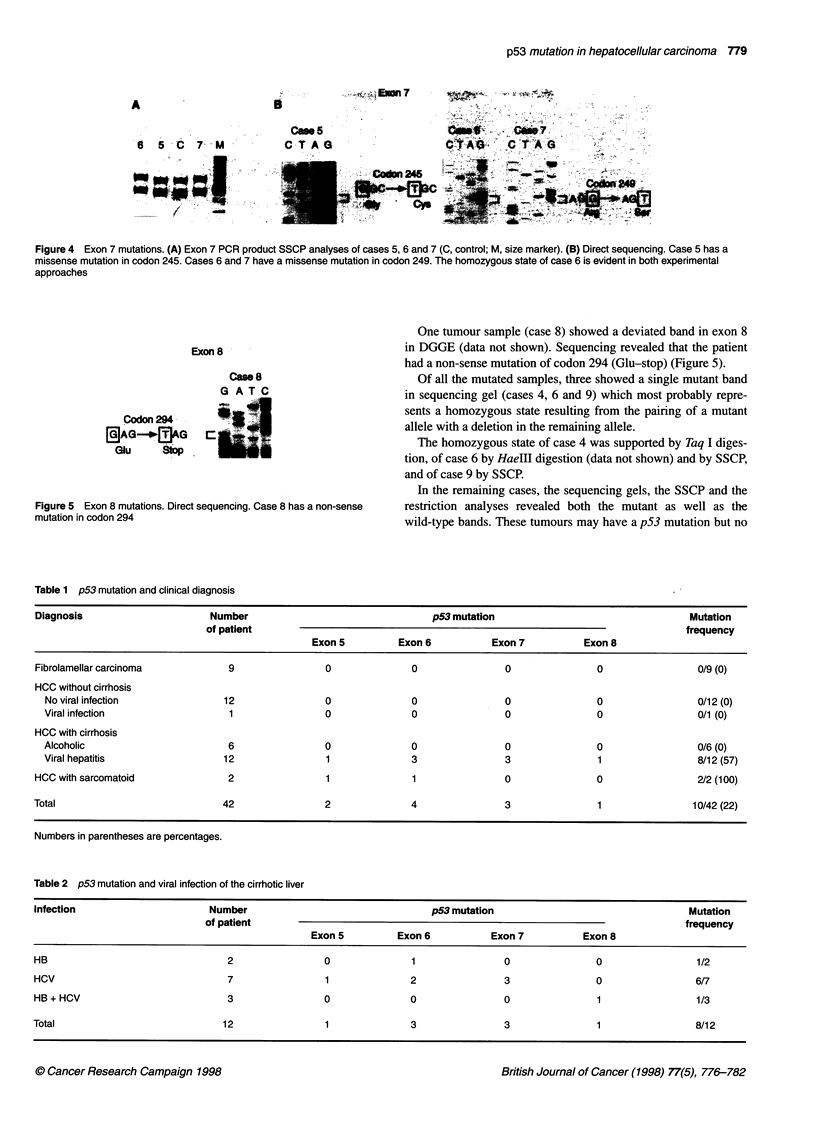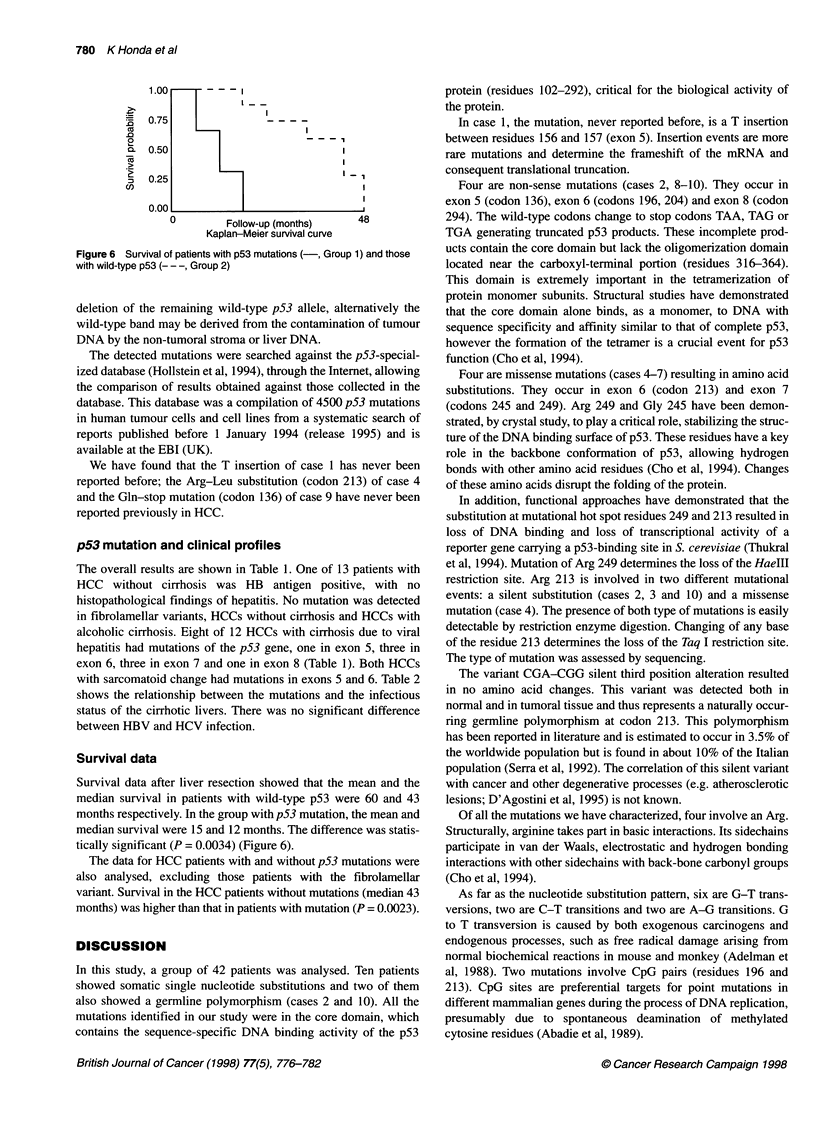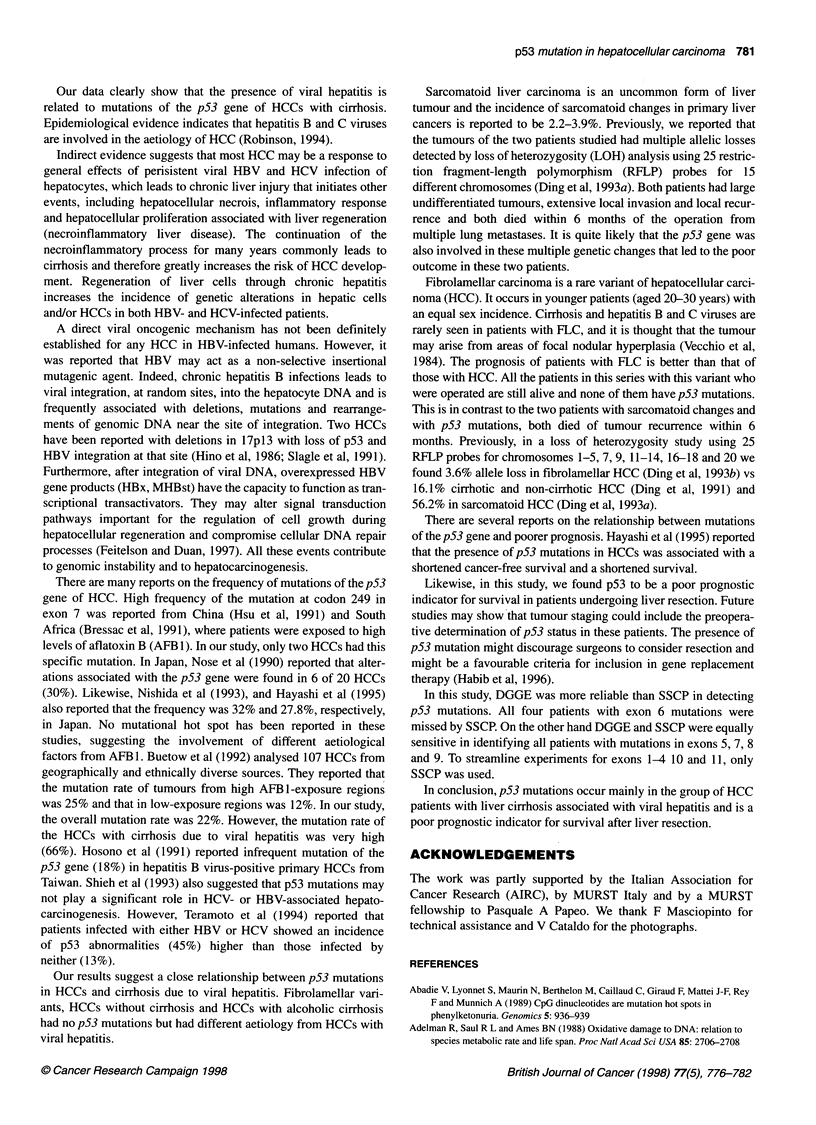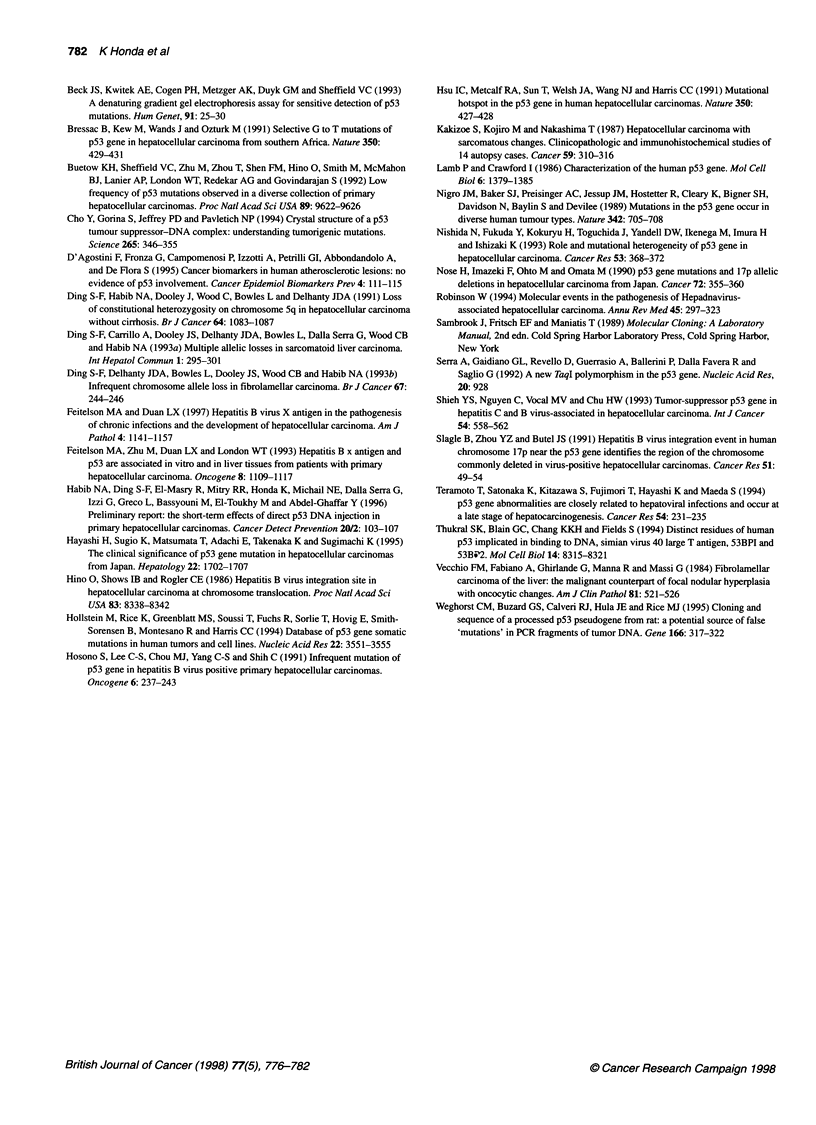# p53 mutation is a poor prognostic indicator for survival in patients with hepatocellular carcinoma undergoing surgical tumour ablation.

**DOI:** 10.1038/bjc.1998.126

**Published:** 1998-03

**Authors:** K. Honda, E. SbisÃ , A. Tullo, P. A. Papeo, C. Saccone, S. Poole, M. Pignatelli, R. R. Mitry, S. Ding, A. Isla, A. Davies, N. A. Habib

**Affiliations:** Department of Surgery, Hammersmith Hospital, RPMS, London, UK.

## Abstract

**Images:**


British Journal of Cancer (1998) 77(5), 776–782
© 1998 Cancer Research Campaign

### *p53* mutation is a poor prognostic indicator for survival in patients with hepatocellular carcinoma undergoing surgical tumour ablation

**K Honda[1], E Sbisà[2], A Tullo[2], PA Papeo[3], C Saccone[3], S Poole[2], M Pignatelli[1], RR Mitry[1], S Ding[1], A Isla[1], A Davies[1] and NA Habib[1]**

[1]Departments of Surgery and Pathology, Hammersmith Hospital, RPMS, London, UK; [2]Centro di Studio sui Mitocondri e Metabolismo Energetico, CNR Bari, via Amendola 165A, 70126 Bari, Italy; [3]Department of Biochemistry and Molecular Biology, University of Bari, via Orabona 4, 70126 Bari, Italy

**Summary** Forty-two patients with hepatocellular carcinoma (HCC) were resected and their tumours were analysed for *p53* mutations by GC-clamped denaturing gradient gel electrophoresis (DGGE), single-strand conformation polymorphism (SSCP) and gene sequencing. All the exons have been analysed in this study. Eight of 12 HCCs with cirrhosis due to viral hepatitis and the two patients with sarcomatoid changes displayed *p53* mutations. In contrast, no mutation was observed in the fibrolamellar variant ($n$ = 9), non-cirrhotics ($n$ = 13) and alcoholic cirrhosis ($n$ = 6). The mutations observed were in exons 5–8. Two mutations were observed in codons 136 and 213 as well as a T insertion between residues 156 and 157 (exon 5) and these are reported for the first time in HCC. Likewise, the silent mutation polymorphism in codon 213 was noticed in 3 of the 42 patients. Survival analysis of these patients after surgery showed the mean and median survival in patients with wild-type *p53* to be 60 and 43 months respectively. In the group with *p53* mutations, the mean and median survival was 15 and 12 months. The difference was statistically significant ($P$ = 0.003).

**Keywords:** liver carcinoma; oncosuppressor gene; hepatitis; cirrhosis p53 protein is a DNA-binding, cell-regulating transcription factor that has multiple critical roles in the complex pathway governing cell cycle and in the balance between cell division and apoptosis. Losses and/or mutations of p53 play a crucial role in a large number of malignancies.

The *p53* gene is the most commonly mutated tumour-suppressor gene in various human cancers (Nigro et al, 1989), and it has been reported in hepatocellular carcinomas (HCCs). However, most of these reports studied patients from east Asian countries and Africa, where viral hepatitis and aflatoxins are rather prevalent in contrast to Western countries. We examined 42 primary liver carcinoma specimens, which were surgically resected at the Hammersmith Hospital (UK) with histologically confirmed diagnosis, for mutation of the *p53* gene by denaturing gradient gel electrophoresis (DGGE), single-strand conformation polymorphism (SSCP) and sequence analysis.

The aim of this investigation was to study the presence of *p53* mutation in HCC patients without viral hepatitis, such as those with the fibrolamellar variant, alcoholic cirrhosis or the classical non-cirrhotic HCC patients. In addition, we aimed to find out whether p53 mutation could influence survival after surgical resection in these patients.

*Received 12 February 1997*
*Revised 25 July 1997*
*Accepted 4 August 1997*

*Correspondence to:* NA Habib, Department of Surgery, Royal Postgraduate Medical School, Du Cane Road, London W12 0NN, UK

### PATIENTS AND METHODS

Forty-two patients with HCC were studied, of whom nine had the fibrolamellar variant of HCC. Eighteen of the 42 HCC patients had underlying cirrhosis (six alcoholic, 12 non-alcoholic). The 12 patients with non-alcoholic cirrhosis had serological evidence of viral infection (two were HBs antigen and HcVAb positive, seven were HCV antibody positive and three were HBs antigen and HCV antibody positive). The remaining 13 had non-cirrhotic HCC tumours. A further two patients had sarcomatoid transformation of hepatocellular carcinoma (Kakizoe et al, 1987). The study included 14 women and 28 men with a mean age of 54.6 years (range 14–73 years). All patients underwent surgical resection of their tumours. None of the patients had any preoperative chemotherapy. Tumour tissue samples as well as surrounding non-tumorous liver tissue samples were obtained at the time of operation. The tissue samples were fixed in 10% neutral formalin and subjected to histopathological examination. A portion of each specimen was frozen in liquid nitrogen immediately after resection and stored at – 70°C until DNA extraction. DNA was prepared from tissue specimens by standard phenol–chloroform methods (Sambrook et al, 1989).

### PCR amplification for the DGGE study

Primers flanking p53 exon sequences were prepared according to the previous report (Beck et al, 1993). One microgram of genomic DNA was mixed with 50 pmol of each appropriate oligonucleotide primer and with 0.2 mmol $l^{-1}$ of each deoxyribonucleotide triphosphate and 1.5 units of *Taq* DNA polymerase (Bioline), in 50 µl of

The three first authors had equal contribution to this study.

**Figure 1** Exon 5 mutation 5. **(A)** Exon 5 PCR product SSCP analysis of cases 1 and 9 (C, control). **(B)** Direct sequencing. The T insertion (case 1) determines the shift of the sequencing pattern of the mutated allele (compare normal and tumoral sequences). In case 9, a C → T transition produces a non-sense mutation. The homozygous state of case 9 is evident in both experimental approaches its standard 'potassium chloride' buffer. Samples were incubated in a DNA thermal cycler (GeneAmp PCR system 2400, Perkin-Elmer) for a total of 40 cycles at 94°C, 58°C, 72°C for 30 s at each temperature. Four microlitres of the polymerase chain reaction (PCR) products were subjected to electrophoresis on a 2% agarose gel to examine successful amplification of each fragment.

### DGGE

The optimum gradient for each PCR product was determined with perpendicular DGGE according to the manufacturer's instructions (D-Gene Denaturing Gel Electrophoresis System, Bio-Rad). The ranges of denaturant of parallel DGGE and the optimum conditions of electrophoresis were previously reported (Beck et al, 1993). Gels were stained with ethidium bromide and were photographed using Polaroid film.

### PCR and single-strand conformation polymorphism analysis

Primers for DNA amplification of the complete coding region, including exon–intron junctions, were appropriately selected from the human *p53* gene sequence (Lamb and Crawford, 1986).

The reaction mixture contained 100 ng of genomic DNA, 30 pmol of appropriate oligonucleotide primer pairs, 0.1 mM dNTPs, 50 mM potassium chloride, 1.5 mM magnesium chloride, 10 mM Tris-HCl pH 9, 2.5 U of *Taq* DNA Pol (Pharmacia Biotech) in a total volume of 100 µl. In each set of PCR reactants, a negative no-template control was included. The amplified products were purified to eliminate primers and non-specific products on 2% agarose Tris–borate–EDTA (TBE) gel. The appropriate band was cut out from the gel and eluted by QIAquick Gel Extraction kit (Qiagen).

Five microlitres of PCR product (300 ng) were mixed with 2 µl of 1% sodium deoxycholate, 2 µl of EDTA 0.11 M pH 7.5 and 5 µl of loading buffer (deionized formamide 98%, 2 µl of EDTA 0.11 µl of bromophenol blue–xylene cyanol 0.3%). The samples were boiled for 5 min and immediately subjected to electrophoresis on non-denaturing polyacrylamide gel; 8 × 10 cm 0.75-thick polyacrylamide gels and 0.5 × TBE pH 7.5 running buffer were used. A constant internal buffer temperature was maintained during the gel run. Gels were run at 200 V for 1–4 h, depending on the fragment size and gel percentage.

After electrophoresis, the gels were silver stained and dried down.

### Codon 213 and 249 restriction analyses

An aliquot of exon 6 PCR product was digested with *Taq* I restriction endonuclease at 65°C for 2 h to identify 213 mutation. Substitution of any bases of codon 213 results in the loss of a *Taq* I site, while the PCR product of the wild type is digested into 140- and 96-bp fragments.

An aliquot of exon 7 PCR product was digested with *Hae*III at 37°C for 2 h to identify 249 mutation (data not shown).

Manufacturer-provided buffers and instructions were used. The digested DNAs were electrophoresed on a 1.5% agarose gel and the DNA was visualized by ethidium bromide staining.

### Sequence

Exons showing an altered migration in SSCP and DGGE were sequenced both with forward and reverse primers to confirm the mutations on both strands. When the tumour was identified to have a mutation, the p53 sequence of the non-tumorous liver tissue from the same individual was also evaluated. Sequence compression problems, as a result of the high G-C content in the gene, were solved by using modified nucleotides, such as deazanucleotides and inosine and controlled temperature conditions in electrophoretic running.

Sequencing primers were the same as those used for SSCP. A cycle-sequencing kit (Perkin Elmer) and [$^{35}$Sd]ATP were used for sequencing. The products reaction was electrophoresed on a denaturing 6% polyacrylamide–7 M urea gel according to the manufacturer's instructions.

The survival data of all these patients after hepatectomy were collected prospectively and were analysed using the log-rank test.

### RESULTS

Two patients (cases 1 and 9) had mutation in exon 5 as shown in the SSCP analysis (Figure 1A). Direct sequencing showed that case no. 1 had a T insertion between residues 156 and 157 (Figure 1B). Sequencing analysis of case no. 9 showed a mutation of codon 136, CAA–TAA with a substitution from glutamine to a stop codon (Figure 1B). The tumour of this patient had sarcomatoid change, which is thought to be a more aggressive variant of HCC.

DGGE analysis of exon 6 of four patients (cases 2, 3, 4 and 10) showed in the tumoral samples a complex deviated pattern compared with the control (Figure 2). Case 4 had deviated bands

© Cancer Research Campaign 1998

**Figure 2** Exon 6 mutations. DGGE in tumour **(A)** and non-tumoral tissue **(B)** in cases 2, 3, 4 and 10 showing mutations and constitutional polymorphism (wt, wild type)

**Figure 3** Exon 6 mutations. **(A)** Codon 213 restriction analysis. *Taq* I-restricted exon 6 PCR products (236 bp) of cases 10, 2, 3 and 4. C, Control; B, blood, T, tumoral, N, non-tumoral. *Taq* I digests the wild-type (wt) DNA into two fragments of 140 and 96 bp. Substitution of any bases in the codon 213 determines the loss of the restriction site. Cases 10, 2 and 3 have a germline polymorphism, present in tumoral and non-tumoral DNA. Case 4 has a homozygous mutation in tumoral DNA. The type of mutation is assessed by sequencing. **(B)** Codon 213 direct sequencing analysis. Case 3: the germline polymorphism is a silent substitution of the third codon position. The same substitution is present in cases 10 and 2 (sequencing data not shown). Case 4: the mutation is a missense mutation of the second codon position. **(C)** Direct sequencing. Cases 10 and 2 have a non-sense mutation in codon 204 and 196, respectively, beside the 213 germline polymorphism only in the tumoral tissue. Whereas cases 2, 3 and 10 showed in the non-tumoral liver samples some of the deviated bands that were present in their tumoral counterparts. Sequencing analysis revealed that all four patients had a mutation in codon 213. The sequencing of tumoral and non-tumoral tissues of cases 2, 3 and 10 revealed a germline polymorphism due to a silent substitution of the third position of codon 213 (CGA–CGG) resulting in no amino acid changes (Figure 3B). Case 4 had a missense mutation (Arg–Leu) due to a G–T transversion in the second position of codon 213 (CGA–CTA). Mutation of any base of codon 213 results in the loss of the *Taq* I site. As shown in Figure 3A, cases 2, 3 and 10 had a heterozygous germline substitution both in the tumoral and non-tumoral tissue. Case 4, instead, had a homozygous mutation only in the tumoral liver.

The sequencing of cases 2 and 10 revealed two non-sense mutations in the tumoral tissue at codon 196 (Arg → stop) and 204 (Glu → stop), respectively (Figure 3C), besides the 213 polymorphism.

As for exon 7, three tumour samples (cases 5–7) showed deviated bands in SSCP analysis (Figure 4A). HaeIII analysis revealed that two of them (cases 6 and 7) had codon 249 mutation after the loss of the restriction site (data not shown). Sequence analysis showed a missense mutation AGG–AGT leading to amino acid substitution from arginine to serine (Figure 4B). Sequencing and *Hae*III digestion suggest that case 6 had a mutation in the homozygous state. Case 5 had a missense mutation GGC–TGC leading to amino acid substitution from glycine to cysteine (Figure 4B).

 © Cancer Research Campaign 1998

**Figure 4** Exon 7 mutations. **(A)** Exon 7 PCR product SSCP analyses of cases 5, 6 and 7 (C, control; M, size marker). **(B)** Direct sequencing. Case 5 has a missense mutation in codon 245. Cases 6 and 7 have a missense mutation in codon 249. The homozygous state of case 6 is evident in both experimental approaches

**Figure 5** Exon 8 mutations. Direct sequencing. Case 8 has a non-sense mutation in codon 294

One tumour sample (case 8) showed a deviated band in exon 8 in DGGE (data not shown). Sequencing revealed that the patient had a non-sense mutation of codon 294 (Glu–stop) (Figure 5).

Of all the mutated samples, three showed a single mutant band in sequencing gel (cases 4, 6 and 9) which most probably represents a homozygous state resulting from the pairing of a mutant allele with a deletion in the remaining allele.

The homozygous state of case 4 was supported by *Taq* I digestion, of case 6 by *Hae*III digestion (data not shown) and by SSCP, and of case 9 by SSCP.

In the remaining cases, the sequencing gels, the SSCP and the restriction analyses revealed both the mutant as well as the wild-type bands. These tumours may have a *p53* mutation but no

**Table 1** *p53* mutation and clinical diagnosis

| Diagnosis | Number of patient | *p53* mutation | | | | Mutation frequency |
|---|---|---|---|---|---|---|
| | | Exon 5 | Exon 6 | Exon 7 | Exon 8 | |
| Fibrolamellar carcinoma | 9 | 0 | 0 | 0 | 0 | 0/9 (0) |
| HCC without cirrhosis | | | | | | |
| No viral infection | 12 | 0 | 0 | 0 | 0 | 0/12 (0) |
| Viral infection | 1 | 0 | 0 | 0 | 0 | 0/1 (0) |
| HCC with cirrhosis | | | | | | |
| Alcoholic | 6 | 0 | 0 | 0 | 0 | 0/6 (0) |
| Viral hepatitis | 12 | 1 | 3 | 3 | 1 | 8/12 (57) |
| HCC with sarcomatoid | 2 | 1 | 1 | 0 | 0 | 2/2 (100) |
| Total | 42 | 2 | 4 | 3 | 1 | 10/42 (22) |

Numbers in parentheses are percentages.

**Table 2** *p53* mutation and viral infection of the cirrhotic liver

| Infection | Number of patient | *p53* mutation | | | | Mutation frequency |
|---|---|---|---|---|---|---|
| | | Exon 5 | Exon 6 | Exon 7 | Exon 8 | |
| HB | 2 | 0 | 1 | 0 | 0 | 1/2 |
| HCV | 7 | 1 | 2 | 3 | 0 | 6/7 |
| HB + HCV | 3 | 0 | 0 | 0 | 1 | 1/3 |
| Total | 12 | 1 | 3 | 3 | 1 | 8/12 |

© Cancer Research Campaign 1998 

**Figure 6** Survival of patients with p53 mutations (—, Group 1) and those with wild-type p53 (- - -, Group 2)

deletion of the remaining wild-type *p53* allele, alternatively the wild-type band may be derived from the contamination of tumour DNA by the non-tumoral stroma or liver DNA.

The detected mutations were searched against the *p53*-specialized database (Hollstein et al, 1994), through the Internet, allowing the comparison of results obtained against those collected in the database. This database was a compilation of 4500 *p53* mutations in human tumour cells and cell lines from a systematic search of reports published before 1 January 1994 (release 1995) and is available at the EBI (UK).

We have found that the T insertion of case 1 has never been reported before; the Arg–Leu substitution (codon 213) of case 4 and the Gln–stop mutation (codon 136) of case 9 have never been reported previously in HCC.

### *p53* mutation and clinical profiles

The overall results are shown in Table 1. One of 13 patients with HCC without cirrhosis was HB antigen positive, with no histopathological findings of hepatitis. No mutation was detected in fibrolamellar variants, HCCs without cirrhosis and HCCs with alcoholic cirrhosis. Eight of 12 HCCs with cirrhosis due to viral hepatitis had mutations of the *p53* gene, one in exon 5, three in exon 6, three in exon 7 and one in exon 8 (Table 1). Both HCCs with sarcomatoid change had mutations in exons 5 and 6. Table 2 shows the relationship between the mutations and the infectious status of the cirrhotic livers. There was no significant difference between HBV and HCV infection.

### Survival data

Survival data after liver resection showed that the mean and the median survival in patients with wild-type p53 were 60 and 43 months respectively. In the group with *p53* mutation, the mean and median survival were 15 and 12 months. The difference was statistically significant ($P = 0.0034$) (Figure 6).

The data for HCC patients with and without *p53* mutations were also analysed, excluding those patients with the fibrolamellar variant. Survival in the HCC patients without mutations (median 43 months) was higher than that in patients with mutation ($P = 0.0023$).

### DISCUSSION

In this study, a group of 42 patients was analysed. Ten patients showed somatic single nucleotide substitutions and two of them also showed a germline polymorphism (cases 2 and 10). All the mutations identified in our study were in the core domain, which contains the sequence-specific DNA binding activity of the p53 protein (residues 102–292), critical for the biological activity of the protein.

In case 1, the mutation, never reported before, is a T insertion between residues 156 and 157 (exon 5). Insertion events are more rare mutations and determine the frameshift of the mRNA and consequent translational truncation.

Four are non-sense mutations (cases 2, 8–10). They occur in exon 5 (codon 136), exon 6 (codons 196, 204) and exon 8 (codon 294). The wild-type codons change to stop codons TAA, TAG or TGA generating truncated p53 products. These incomplete products contain the core domain but lack the oligomerization domain located near the carboxyl-terminal portion (residues 316–364). This domain is extremely important in the tetramerization of protein monomer subunits. Structural studies have demonstrated that the core domain alone binds, as a monomer, to DNA with sequence specificity and affinity similar to that of complete p53, however the formation of the tetramer is a crucial event for p53 function (Cho et al, 1994).

Four are missense mutations (cases 4–7) resulting in amino acid substitutions. They occur in exon 6 (codon 213) and exon 7 (codons 245 and 249). Arg 249 and Gly 245 have been demonstrated, by crystal study, to play a critical role, stabilizing the structure of the DNA binding surface of p53. These residues have a key role in the backbone conformation of p53, allowing hydrogen bonds with other amino acid residues (Cho et al, 1994). Changes of these amino acids disrupt the folding of the protein.

In addition, functional approaches have demonstrated that the substitution at mutational hot spot residues 249 and 213 resulted in loss of DNA binding and loss of transcriptional activity of a reporter gene carrying a p53-binding site in *S. cerevisiae* (Thukral et al, 1994). Mutation of Arg 249 determines the loss of the *Hae*III restriction site. Arg 213 is involved in two different mutational events: a silent substitution (cases 2, 3 and 10) and a missense mutation (case 4). The presence of both type of mutations is easily detectable by restriction enzyme digestion. Changing of any base of the residue 213 determines the loss of the *Taq* I restriction site. The type of mutation was assessed by sequencing.

The variant CGA–CGG silent third position alteration resulted in no amino acid changes. This variant was detected both in normal and in tumoral tissue and thus represents a naturally occurring germline polymorphism at codon 213. This polymorphism has been reported in literature and is estimated to occur in 3.5% of the worldwide population but is found in about 10% of the Italian population (Serra et al, 1992). The correlation of this silent variant with cancer and other degenerative processes (e.g. atherosclerotic lesions; D'Agostini et al, 1995) is not known.

Of all the mutations we have characterized, four involve an Arg. Structurally, arginine takes part in basic interactions. Its sidechains participate in van der Waals, electrostatic and hydrogen bonding interactions with other sidechains with back-bone carbonyl groups (Cho et al, 1994).

As far as the nucleotide substitution pattern, six are G–T transversions, two are C–T transitions and two are A–G transitions. G to T transversion is caused by both exogenous carcinogens and endogenous processes, such as free radical damage arising from normal biochemical reactions in mouse and monkey (Adelman et al, 1988). Two mutations involve CpG pairs (residues 196 and 213). CpG sites are preferential targets for point mutations in different mammalian genes during the process of DNA replication, presumably due to spontaneous deamination of methylated cytosine residues (Abadie et al, 1989).

 © Cancer Research Campaign 1998

Our data clearly show that the presence of viral hepatitis is related to mutations of the *p53* gene of HCCs with cirrhosis. Epidemiological evidence indicates that hepatitis B and C viruses are involved in the aetiology of HCC (Robinson, 1994).

Indirect evidence suggests that most HCC may be a response to general effects of perisistent viral HBV and HCV infection of hepatocytes, which leads to chronic liver injury that initiates other events, including hepatocellular necrois, inflammatory response and hepatocellular proliferation associated with liver regeneration (necroinflammatory liver disease). The continuation of the necroinflammatory process for many years commonly leads to cirrhosis and therefore greatly increases the risk of HCC development. Regeneration of liver cells through chronic hepatitis increases the incidence of genetic alterations in hepatic cells and/or HCCs in both HBV- and HCV-infected patients.

A direct viral oncogenic mechanism has not been definitely established for any HCC in HBV-infected humans. However, it was reported that HBV may act as a non-selective insertional mutagenic agent. Indeed, chronic hepatitis B infections leads to viral integration, at random sites, into the hepatocyte DNA and is frequently associated with deletions, mutations and rearrangements of genomic DNA near the site of integration. Two HCCs have been reported with deletions in 17p13 with loss of p53 and HBV integration at that site (Hino et al, 1986; Slagle et al, 1991). Furthermore, after integration of viral DNA, overexpressed HBV gene products (HBx, MHBst) have the capacity to function as transcriptional transactivators. They may alter signal transduction pathways important for the regulation of cell growth during hepatocellular regeneration and compromise cellular DNA repair processes (Feitelson and Duan, 1997). All these events contribute to genomic instability and to hepatocarcinogenesis.

There are many reports on the frequency of mutations of the *p53* gene of HCC. High frequency of the mutation at codon 249 in exon 7 was reported from China (Hsu et al, 1991) and South Africa (Bressac et al, 1991), where patients were exposed to high levels of aflatoxin B (AFB1). In our study, only two HCCs had this specific mutation. In Japan, Nose et al (1990) reported that alterations associated with the *p53* gene were found in 6 of 20 HCCs (30%). Likewise, Nishida et al (1993), and Hayashi et al (1995) also reported that the frequency was 32% and 27.8%, respectively, in Japan. No mutational hot spot has been reported in these studies, suggesting the involvement of different aetiological factors from AFB1. Buetow et al (1992) analysed 107 HCCs from geographically and ethnically diverse sources. They reported that the mutation rate of tumours from high AFB1-exposure regions was 25% and that in low-exposure regions was 12%. In our study, the overall mutation rate was 22%. However, the mutation rate of the HCCs with cirrhosis due to viral hepatitis was very high (66%). Hosono et al (1991) reported infrequent mutation of the *p53* gene (18%) in hepatitis B virus-positive primary HCCs from Taiwan. Shieh et al (1993) also suggested that p53 mutations may not play a significant role in HCV- or HBV-associated hepatocarcinogenesis. However, Teramoto et al (1994) reported that patients infected with either HBV or HCV showed an incidence of p53 abnormalities (45%) higher than those infected by neither (13%).

Our results suggest a close relationship between *p53* mutations in HCCs and cirrhosis due to viral hepatitis. Fibrolamellar variants, HCCs without cirrhosis and HCCs with alcoholic cirrhosis had no *p53* mutations but had different aetiology from HCCs with viral hepatitis.

Sarcomatoid liver carcinoma is an uncommon form of liver tumour and the incidence of sarcomatoid changes in primary liver cancers is reported to be 2.2–3.9%. Previously, we reported that the tumours of the two patients studied had multiple allelic losses detected by loss of heterozygosity (LOH) analysis using 25 restriction fragment-length polymorphism (RFLP) probes for 15 different chromosomes (Ding et al, 1993*a*). Both patients had large undifferentiated tumours, extensive local invasion and local recurrence and both died within 6 months of the operation from multiple lung metastases. It is quite likely that the *p53* gene was also involved in these multiple genetic changes that led to the poor outcome in these two patients.

Fibrolamellar carcinoma is a rare variant of hepatocellular carcinoma (HCC). It occurs in younger patients (aged 20–30 years) with an equal sex incidence. Cirrhosis and hepatitis B and C viruses are rarely seen in patients with FLC, and it is thought that the tumour may arise from areas of focal nodular hyperplasia (Vecchio et al, 1984). The prognosis of patients with FLC is better than that of those with HCC. All the patients in this series with this variant who were operated are still alive and none of them have *p53* mutations. This is in contrast to the two patients with sarcomatoid changes and with *p53* mutations, both died of tumour recurrence within 6 months. Previously, in a loss of heterozygosity study using 25 RFLP probes for chromosomes 1–5, 7, 9, 11–14, 16–18 and 20 we found 3.6% allele loss in fibrolamellar HCC (Ding et al, 1993*b*) vs 16.1% cirrhotic and non-cirrhotic HCC (Ding et al, 1991) and 56.2% in sarcomatoid HCC (Ding et al, 1993*a*).

There are several reports on the relationship between mutations of the *p53* gene and poorer prognosis. Hayashi et al (1995) reported that the presence of *p53* mutations in HCCs was associated with a shortened cancer-free survival and a shortened survival.

Likewise, in this study, we found p53 to be a poor prognostic indicator for survival in patients undergoing liver resection. Future studies may show that tumour staging could include the preoperative determination of *p53* status in these patients. The presence of *p53* mutation might discourage surgeons to consider resection and might be a favourable criteria for inclusion in gene replacement therapy (Habib et al, 1996).

In this study, DGGE was more reliable than SSCP in detecting *p53* mutations. All four patients with exon 6 mutations were missed by SSCP. On the other hand DGGE and SSCP were equally sensitive in identifying all patients with mutations in exons 5, 7, 8 and 9. To streamline experiments for exons 1–4 10 and 11, only SSCP was used.

In conclusion, *p53* mutations occur mainly in the group of HCC patients with liver cirrhosis associated with viral hepatitis and is a poor prognostic indicator for survival after liver resection.

### ACKNOWLEDGEMENTS

The work was partly supported by the Italian Association for Cancer Research (AIRC), by MURST Italy and by a MURST fellowship to Pasquale A Papeo. We thank F Masciopinto for technical assistance and V Cataldo for the photographs.

#### REFERENCES

Abadie V, Lyonnet S, Maurin N, Berthelon M, Caillaud C, Giraud F, Mattei J-F, Rey F and Munnich A (1989) CpG dinucleotides are mutation hot spots in phenylketonuria. *Genomics* **5**: 936–939

Adelman R, Saul R L and Ames BN (1988) Oxidative damage to DNA: relation to species metabolic rate and life span. *Proc Natl Acad Sci USA* **85**: 2706–2708

© Cancer Research Campaign 1998

Beck JS, Kwitek AE, Cogen PH, Metzger AK, Duyk GM and Sheffield VC (1993) A denaturing gradient gel electrophoresis assay for sensitive detection of p53 mutations. *Hum Genet*, **91**: 25–30

Bressac B, Kew M, Wands J and Ozturk M (1991) Selective G to T mutations of p53 gene in hepatocellular carcinoma from southern Africa. *Nature* **350**: 429–431

Buetow KH, Sheffield VC, Zhu M, Zhou T, Shen FM, Hino O, Smith M, McMahon BJ, Lanier AP, London WT, Redekar AG and Govindarajan S (1992) Low frequency of p53 mutations observed in a diverse collection of primary hepatocellular carcinomas. *Proc Natl Acad Sci USA* **89**: 9622–9626

Cho Y, Gorina S, Jeffrey PD and Pavletich NP (1994) Crystal structure of a p53 tumour suppressor–DNA complex: understanding tumorigenic mutations. *Science* **265**: 346–355

D'Agostini F, Fronza G, Campomenosi P, Izzotti A, Petrilli GI, Abbondandolo A, and De Flora S (1995) Cancer biomarkers in human atherosclerotic lesions: no evidence of p53 involvement. *Cancer Epidemiol Biomarkers Prev* **4**: 111–115

Ding S-F, Habib NA, Dooley J, Wood C, Bowles L and Delhanty JDA (1991) Loss of constitutional heterozygosity on chromosome 5q in hepatocellular carcinoma without cirrhosis. *Br J Cancer* **64**: 1083–1087

Ding S-F, Carrillo A, Dooley JS, Delhanty JDA, Bowles L, Dalla Serra G, Wood CB and Habib NA (1993*a*) Multiple allelic losses in sarcomatoid liver carcinoma. *Int Hepatol Commun* **1**: 295–301

Ding S-F, Delhanty JDA, Bowles L, Dooley JS, Wood CB and Habib NA (1993*b*) Infrequent chromosome allele loss in fibrolamellar carcinoma. *Br J Cancer* **67**: 244–246

Feitelson MA and Duan LX (1997) Hepatitis B virus X antigen in the pathogenesis of chronic infections and the development of hepatocellular carcinoma. *Am J Pathol* **4**: 1141–1157

Feitelson MA, Zhu M, Duan LX and London WT (1993) Hepatitis B x antigen and p53 are associated in vitro and in liver tissues from patients with primary hepatocellular carcinoma. *Oncogene* **8**: 1109–1117

Habib NA, Ding S-F, El-Masry R, Mitry RR, Honda K, Michail NE, Dalla Serra G, Izzi G, Greco L, Bassyouni M, El-Toukhy M and Abdel-Ghaffar Y (1996) Preliminary report: the short-term effects of direct p53 DNA injection in primary hepatocellular carcinomas. *Cancer Detect Prevention* **20/2**: 103–107

Hayashi H, Sugio K, Matsumata T, Adachi E, Takenaka K and Sugimachi K (1995) The clinical significance of p53 gene mutation in hepatocellular carcinomas from Japan. *Hepatology* **22**: 1702–1707

Hino O, Shows IB and Rogler CE (1986) Hepatitis B virus integration site in hepatocellular carcinoma at chromosome translocation. *Proc Natl Acad Sci USA* **83**: 8338–8342

Hollstein M, Rice K, Greenblatt MS, Soussi T, Fuchs R, Sorlie T, Hovig E, Smith-Sorensen B, Montesano R and Harris CC (1994) Database of p53 gene somatic mutations in human tumors and cell lines. *Nucleic Acid Res* **22**: 3551–3555

Hosono S, Lee C-S, Chou MJ, Yang C-S and Shih C (1991) Infrequent mutation of p53 gene in hepatitis B virus positive primary hepatocellular carcinomas. *Oncogene* **6**: 237–243

Hsu IC, Metcalf RA, Sun T, Welsh JA, Wang NJ and Harris CC (1991) Mutational hotspot in the p53 gene in human hepatocellular carcinomas. *Nature* **350**: 427–428

Kakizoe S, Kojiro M and Nakashima T (1987) Hepatocellular carcinoma with sarcomatous changes. Clinicopathologic and immunohistochemical studies of 14 autopsy cases. *Cancer* **59**: 310–316

Lamb P and Crawford I (1986) Characterization of the human p53 gene. *Mol Cell Biol* **6**: 1379–1385

Nigro JM, Baker SJ, Preisinger AC, Jessup JM, Hostetter R, Cleary K, Bigner SH, Davidson N, Baylin S and Devilee (1989) Mutations in the p53 gene occur in diverse human tumour types. *Nature* **342**: 705–708

Nishida N, Fukuda Y, Kokuryu H, Toguchida J, Yandell DW, Ikenega M, Imura H and Ishizaki K (1993) Role and mutational heterogeneity of p53 gene in hepatocellular carcinoma. *Cancer Res* **53**: 368–372

Nose H, Imazeki F, Ohto M and Omata M (1990) p53 gene mutations and 17p allelic deletions in hepatocellular carcinoma from Japan. *Cancer* **72**: 355–360

Robinson W (1994) Molecular events in the pathogenesis of Hepadnavirus-associated hepatocellular carcinoma. *Annu Rev Med* **45**: 297–323

Sambrook J, Fritsch EF and Maniatis T (1989) *Molecular Cloning: A Laboratory Manual,* 2nd edn. Cold Spring Harbor Laboratory Press, Cold Spring Harbor, New York

Serra A, Gaidiano GL, Revello D, Guerrasio A, Ballerini P, Dalla Favera R and Saglio G (1992) A new *Taq*I polymorphism in the p53 gene. *Nucleic Acid Res*, **20**: 928

Shieh YS, Nguyen C, Vocal MV and Chu HW (1993) Tumor-suppressor p53 gene in hepatitis C and B virus-associated in hepatocellular carcinoma. *Int J Cancer* **54**: 558–562

Slagle B, Zhou YZ and Butel JS (1991) Hepatitis B virus integration event in human chromosome 17p near the p53 gene identifies the region of the chromosome commonly deleted in virus-positive hepatocellular carcinomas. *Cancer Res* **51**: 49–54

Teramoto T, Satonaka K, Kitazawa S, Fujimori T, Hayashi K and Maeda S (1994) p53 gene abnormalities are closely related to hepatoviral infections and occur at a late stage of hepatocarcinogenesis. *Cancer Res* **54**: 231–235

Thukral SK, Blain GC, Chang KKH and Fields S (1994) Distinct residues of human p53 implicated in binding to DNA, simian virus 40 large T antigen, 53BPI and 53B[illegible]2. *Mol Cell Biol* **14**: 8315–8321

Vecchio FM, Fabiano A, Ghirlande G, Manna R and Massi G (1984) Fibrolamellar carcinoma of the liver: the malignant counterpart of focal nodular hyperplasia with oncocytic changes. *Am J Clin Pathol* **81**: 521–526

Weghorst CM, Buzard GS, Calveri RJ, Hula JE and Rice MJ (1995) Cloning and sequence of a processed p53 pseudogene from rat: a potential source of false 'mutations' in PCR fragments of tumor DNA. *Gene* **166**: 317–322

© Cancer Research Campaign 1998